# Atlanta Academy of Medicine

**Published:** 1871

**Authors:** Edward P. Ingraham

**Affiliations:** Reporter; Atlanta, Ga.


### *Atlanta Academy of Medicine.*

Edw'd P. Ingraham, M.D., Reporter.

*June 30th.*—Dr. Cumming, President, *pro tem.*

Dr. Wells reported a case of wound of the foot, taking on erysipelatous inflammation. Abscess formed at the point of injury. Another abscess appearing in the course of the lymphatics, the glands of the groin enlarged.

Gave chlorate of potash and tinct. chloride of iron in large doses. He cut down on the abscess from which pus was discharged. The wound healed, all erysipelatous inflammation subsided, and the patient was discharged on the fourth day. In such cases, Dr. W. regards tr. ferri. chlo. as the sheet anchor; gives it in large doses, largely diluted.

Dr. Love asked concerning the character of diet upon which the patient had been subsisting—whether animal or vegetable, principally, and whether taken in a state of decomposition?

Dr. W. stated that the patient had every means of obtaining good food, but thought there was a scrofulous tendency in the constitution.

Dr. Banks being called on, reported a case of endo-metritis treated by injections of Lugol's solution of iodine during pregnancy, without producing abortion. The patient had been under the treatment of a number of physicians, and had at different times applications made of nitrate of silver, creosote, and various other remedies, to the os uteri.

On examination, Dr. B. found acute inflammation of an exceedingly small cervical canal; there was a slight sanguineous discharge, with an indurated condition of the cervix. He applied leeches freely every four or five days for two or three weeks, in addition to constitutional treatment; still an indurated condition and catarrhal discharge continued, showing the existence of catarrhal inflammation within the cervix. He then introduced an elm bark tent, replacing it the next day with a sponge tent, until the canal was sufficiently dilated to use injections; five or six drops of Lugol's solution was then injected into the cavity of the uterus.

This treatment was repeated every ten days; after a time (the case improving, though some discharge still continuing), the injections were made less frequently—about every two or three weeks. In the meantime, the patient passed one epoch without the appearance of the menses, and morning sickness supervened. The latter was regarded as sympathetic, pregnancy not being suspected. The injection was used twice after the cessation of the menses, at intervals of two or three weeks. It becoming evident that she was pregnant, this treatment was discontinued. At the expiration of full term, the patient was delivered of a well developed child. That the last two injections did no harm, was proven by the fact that abortion was not produced, the utero-gestation proceeding to full term.

Dr. Harden inquired if Dr. B. passed the instrument used for injecting up to the fundus of the uterus?

Dr. B. replied that he introduced it two and one-third inches into the uterus.

Dr. Cumming—Had the patient been pregnant before?

Dr. B.—She had. Another point of interest that he had neglected to mention was, that the patient always noticed the taste of iodine soon after the applications had been made to the uterus.

Dr. Cumming asked if Dr. B. withdrew the syringe as he forced in the injection?

Dr. B.—I did.

Dr. W. F. Westmoreland inquired of Dr. B. if he had ever met with any trouble from the use of uterine injections?

Dr. B. replied that he had, and that he could not state why; that in *some cases* the injection of fluids into the cavity of the uterus would cause uterine colic; he had met with such cases. It was his custom to first test the irritability of the organ by introducing Sims' sound. If this was tolerated and there was no spasmodic action of the cervix, he proceeded to use injections.

Dr. McDowell enquired what was Dr. B.'s treatment in cases where he found the uterus irritable?

Dr. B. first took into consideration the general health. As a rule they could bear the application of leeches to the os, and its dilatation by means of elm bark or sponge tents. In this way he endeavored gradually to overcome the irritability.

In reply to an inquiry of Dr. Cumming, as to how many leeches he usually applied at once, he stated never more than four, rarely more than three, as he considered there might be danger of hemorrhage and subsequent prostration of the patient from alarm; he thought it better to apply two or three at once, and make the applications at shorter intervals, than too many at the same time. He estimated that one leech, together with the after hemorrhage, removes from two to two and a half ounces of blood, when the os is highly inflamed; if indurated, the quantity will be less.

Dr. W. F. Westmoreland desired to know what preparation Dr. B. found best for injection?

Dr. B. preferred Lugol's solution, undiluted, for injection; as an application to the os and neck, he used the tincture of iodine. When there were abrasions, he used nitrate of silver. Referring to the case he had just reported, he believed that the decidua vera was an exudation from the mucous membrane of the uterus; and during the earlier stages of pregnancy was not a shut sack, but open at the os as well as at the entrance of the fallopian tubes, and would allow the introduction of a syringe through the os; and that in this case he thought the instrument must have passed into the cavity of the decidua, and that that membrane was not pushed before it.

Dr. Wells enquired if it were not possible for the injection to have regurgitated?

Dr. B. did not think it possible, and that he was satisfied that the syringe entered the cavity of the uterus.

Dr. Vance suggested that the case might have been one of double uterus; did not think it possible for an instrument and injection to be introduced into the cavity of a pregnant uterus without injury.

Dr. B. was satisfied there was no double womb, and that he introduced the instrument into the same place every time.

Dr. Vance reported case of a young lady, æt. 16—distortion of face by contraction of muscles, resulting from paralysis of opposite side, occurring in infancy. He divided the zygomatics and levator muscles subcutaneously and drew the whole as much as possible toward the opposite side. Desires information as to the best mode of retaining the face in a most natural position. States that Dr. Garrod used elastics with an eliptical incision of the paralysed muscles. Dr. V. had not tried galvanism in this case, as it was one of sixteen years standing.

Dr. W. F. Westmoreland replied that the great trouble would be, that so soon as union takes place between the divided muscles the contractions will again recur, as in clubfoot, when the tendon has united, unless extension is kept up.

Dr. V. stated that extension had been kept up so long as soreness had existed, when it was discontinued, believing that no more plastic matter would be thrown out thereafter.

Dr. W. replied that the same principle would hold good after as before union. Dr. Sayer holds that relief by incision is only temporary, while he attaches much importance to continued extension, thus to oppose antagonizing muscles.

*July 7th.*—The President, Dr. Logan, in the chair.

Dr. A. W. Griggs being present, was called on and reported the following case: Was called, January last, to see Mr. H., æt 55, suffering from a large tumor, deep seated between umbilicus and ilium of the right side. To the touch it was hard and wenlike, deeply attached, was painless and had existed for a number of years—the patient not being able to urinate except in the position to defecate.

On the 17th of June last, he had had no alvine evacuation for ten days. This had been preceded by a slight diarrhœa.

When seen in January, he had some tympanitis. Examination per rectum revealed a large tumor pressing on the pubes, hard and non-fluctuating. Other physicians in consultation, thinking they could detect fluctuation, the tumor was tapped, which resulted in a slight sanious discharge. The patient became faint, had five or six operations within an hour; vomited, at first, dark, and afterwards a claret colored matter, and died at 6 P. M., the same day. Dr. G. advanced the opinion that the case was one of malignant disease of the kidney. No post-mortem.

Dr. W. F. Westmoreland—Had seen the same case in May previous. To the touch it then conveyed the sensation of an ovarian tumor. After two or three examinations, Dr. W. had decided in his own mind, that it was a tumor connected with the bladder, non-malignant, and not being painful, advised non-interference in his then present condition.

Dr. Vance being called on for a report of his own case, having recently suffered severely, made the following:

He was taken suddenly ill, Friday, May 12th, with a severe pain over the region of the ilio-cœcal valve. Very soon a tumor appeared in this locality, about the size of an orange. A mustard plaster was applied immediately, and a bottle of the solution of citrate of magnesia was taken without any effect whatever. The pain continued until the next morning (13th), when Dr. Logan being called, advised a mild cathartic, which seemed to relieve the pain for the time being, but did not accomplish its object. During the day, about one-sixth of a grain of sulph. of morphia was injected hypodermically, which gave relief for a short time. The same was repeated in the evening.

On Sunday (14th), had a copious watery, evacuation which was followed by transient relief. The pain returned again very soon and gave great distress till the following morning (15th), when a table-spoonful of powdered sulphur was administered. This brought away the first solid fæces since the beginning of the attack.

On the 16th, a blister was applied over the tumor—the pain continuing.

17th. The blister was repeated—the previous one not having drawn well. The tumor remaining the same size. Pain was somewhat relieved after the blistering.

18th. Dr. Logan ordered pil. hydrarg, grs. ii., extr. belladonna, gr. ¼, extr. hyascyamus gr. i., at night. This was repeated for several days. The tumor slowly disappeared, with soreness on pressure. At the end of six weeks it had disappeared.

Dr. Harden stated that he had, twenty years ago, suffered from the same trouble—pain and tumor over ilio-cœcal valve. Active cathartics were used. Still suffers from effects. He advises in such cases, the avoidance of drastic cathartics and calomel. The constipation results from hardened fæces. The object is to induce an evacuation, avoiding nausea and disturbance of the secretions. He advises salines in small doses, to dissolve the fæces—finds senna also to act finely in such cases. Produce an alvine evacuation first, and if the calomel be indicated, use it.

Dr. J. G. Westmoreland had had some experience in what he called impacted cœcum. Had fullness and tenderness over the point. In such cases injections returned bring with them no fæcal odor. He does not see any danger or injurious results from the use of calomel, but uses enemata to evacuate the lower bowel.

Dr. Harden replied that in his cases, he never neglected the use of enemata—had used the elastic tube, but is satisfied he had derived as much or even more benefit from saline purgatives than from anything else.

Dr. J. G. W. had used pumps, and found them sufficient without the tubes.

Dr. Griggs had seen a number of cases of obstruction of bowels, from impacted fæces and from intussusception. Calomel in small doses, he thinks, may not cause nausea; regards salines as good, but relies mainly on enemata; he first used alkalies in solution, follows them by tartaric acid in solution, which produced effervescence and an opperation; had used in this way as many as half a dozen seidlitz powders at once. In a case of constipation of ten day's standing, and stercoraceous vomiting, when other enemata had produced no effect, and when the tube and large quantities of warm water failed, he then resorted to the use of the alkaline bicarbonates and acids in separate solutions, introducing first one, then the other, when copious evacuations followed, and the patient was relieved.

This practice, Dr. G. claimed to have originated with a friend and former partner of his, who relieved several cases in the same way, a reference to which would be found in the works of Wood and of Flint on practice.

Dr. Love reported, in brief, three cases of obstipation, resulting in typhlitis of one, and ultimately in artificial anus in two. One, a child at nine years—obstruction from cherry seed—artificial anus over the region of cœcum. After discharge of foreign substances, the natural passage was again opened, when the artificial passage closed.

The second case, female aged thirty, had been in the habit of eating largely of magnesia, chalk, etc., to arrest acidity, or gratify a morbid appetite originating in deranged digestion. The calcareous substances became impacted or concreted in the cœcum, produced inflammation, obstipation, and ultimately death. Post-mortem revealed a large mass of concrete, filling cœcal end of colon.

The third case, female, colored, æt 26. Case commenced with constipation, extreme pain in cœcal region, and presented the usual concomitants of obstruction. Repeated injections and cathartics, from salts to croton oil were used. Vomiting forbade the use of more medicine by the stomach, when warm water in very large quantity, was thrown up the rectum with pump, giving an uninterrupted stream. This produced an evacuation, when quantities of watermelon seed were discharged. Notwithstanding this, the local pain continued, a tumor formed in ilio-cœcal region, which, at the end of many days pointed; was opened, and an additional quantity of watermelon seed, pus, blood, mucous and fœcal matter were discharged ; contents of bowels were, for a while, discharged by both passages; the artificial one ultimately closed, but the patient succumbed from a low form of fever, in which there was more or less peritonitis.

Dr. Love stated that he had been able to relieve some cases of obstipation by the *steady* and *continuous* introduction through the rectum of warm water, steadying the pressure against the resisting tissues by the steady force on the instrument, but he had never used the acids and alkalies for this purpose, in this way, *per rectum.* By the *stomach* he had resorted to effervescence in three very singular cases, in which the symptoms of poisoning by stramonium, had followed the over engorgement of the stomach with red-skinned yam potatoes, eaten *raw.*

The first case occurred in his practice in the fall of 1847, Negro boy æt 14, taken suddenly ill while gathering potatoes, presented, in brief, symptoms of stramonium poison nearer than anything to which it could be likened, and if he had died, Dr. L. would have believed it a case of death by stramonium. Emetic medicines were used freely of the non-irritating kind. Tart. emet., sulph. zinc, etc., given as much as felt authorized to risk in stomach—irritating the fauces and œsophagus, all to no effect—no contraction of stomach could be induced. An ash-hopper near by suggested the idea of acids and alkalies to "*boil out*" the supposed stramonium. With no time to lose, from the indications, and few means at command, gave a tea-cupful of lye and the little tartaric acid he had *drenched down* followed by another tea-cupful of weak lye and a little soda—all he could get—rolled him over a few times, when he "*boiled* out" several quarts of half-masticated *raw, red, yam potatoes.* Nothing else in the way of food or poison, to account for his condition.

The boy remained insensible twenty-four hours longer—deaf the same length of time, and blind for about eight hours—had some fever on re-action, but made good his recovery.

In 1859, another case precisely similar in every respect, occurred in a boy of about the same age, then the property of Col. H. J. Lamar, of Macon. Under the use of citric acid and bi-carb. pot., one or two table-spoonfulls, dissolved separately and given, first the acid then the alkali, he was relieved rapidly of a *most incredible quantity of coarse, ground, raw, red, yam potatoes.*

Still another case, where watermelons, in addition to the potatoes were taken. Tartaric acid and soda were used in same way, with desired results. The necessities of an emergency prompted this treatment in the outset, he never having seen any notice of the practice in the hands of others. Since then, however, he has found the plan highly recommended by Dr. Frost, of Charleston (and published in his Syllabus.)

Dr. Love strongly urges this plan of emptying the stomach when there exists such complete inertia, and the stomach-pump is not available or not at hand. Whether in these cases the effect was due entirely to the over-distension of the stomach or whether there was a specific effect from the potatoes, has been a question in his mind—he has never seen the same train of symptoms follow over distension by other articles of food, nor by other species of the potato. The wall-climbing, vaulting and other fantastic gymnastics seen in these cases, he has never seen in any others except from the toxication of stramonium.

In all similar cases he would resort at once to the *effervescent use of acids and alkalies.*

Dr. Boring had seen injurious effects from drinking ley, in one case, a gill had proved fatal.

Dr. J. G. Westmoreland doubts if there exists any carbonate in ley, and whether with an acid it would effervesce.

Dr. Stout is of the impression that ley contains carbonates. He thinks that effervesence to empty the stomach should be resorted to with circumspection lest rupture or suffocation should follow. Thinks that there would be less danger in cases among the young and robust. He is struck with the plan of treatment in such cases, but thinks it should not be resorted to rashly.

